# A Conserved Signalling Pathway for Amoebozoan Encystation that was Co-Opted for Multicellular Development

**DOI:** 10.1038/srep09644

**Published:** 2015-04-16

**Authors:** Yoshinori Kawabe, Christina Schilde, Qingyou Du, Pauline Schaap

**Affiliations:** 1 College of Life Sciences, University of Dundee, Dundee DD15EH, Scotland, UK

## Abstract

The evolution of multicellularity required novel mechanisms for intercellular communication, but their origin is unclear. *Dictyostelium* cells exchange signals to position specialized cell types in multicellular spore-bearing structures. These signals activate complex pathways that converge on activation of cAMP-dependent protein kinase (PKA). Genes controlling PKA were detected in the Dictyostelid unicellular ancestors, which like most protists form dormant cysts when experiencing environmental stress. We deleted *PKA* and the adenylate cyclases *AcrA* and *AcgA,* which synthesize cAMP for PKA activation, in the intermediate species *Polysphondylium*, which can develop into either cysts or into multicellular structures. Loss of *PKA* prevented multicellular development, but also completely blocked encystation. Loss of *AcrA* and *AcgA*, both essential for sporulation in *Dictyostelium,* did not affect *Polysphondylium* sporulation, but prevented encystation. We conclude that multicellular cAMP signalling was co-opted from PKA regulation of protist encystation with progressive refunctionalization of pathway components.

The transition from uni- to multicellularity occurred at least eight times independently and enabled the evolution of complex macroscopic life forms. Multicellularity allows division of labour between cells and the construction of multi-layered tissues and organs, in which specialized cells perform different functions. However, multicellularity also requires novel mechanisms for intercellular communication that allow the specialized cell types to differentiate in well-regulated proportions and at appropiate locations. Most unicellular eukaryotes or protists have a simple life cycle in which feeding cells differentiate into a dormant cyst, when faced with starvation or other forms of stress^1^. The physiology and differentiation of protists are therefore mainly regulated by environmental signals. Upon transition to multicellularity their sensory systems must have adapted to enable communication between cells. Because the signal processing systems of protists have been little investigated and the early multicellular forms are long extinct, the early stages in the evolution of multicellularity are not understood.

Dictyostelid social amoebas aggregate to form migrating sorogens, which transform into fruiting bodies, containing up to a million cells. These cells differentiate into spores and four different cell types to carry the spore mass aloft. In the genetic model system *Dictyostelium discoideum*, the mechanisms controlling multicellular development have been intensively investigated, highlighting a dominant role for cAMP throughout the developmental programme. *D. discoideum* aggregation is mediated by secreted cAMP pulses that are produced by the adenylate cyclase ACA and detected by surface cAMP receptors (cARs). Once aggregated, a second adenylate cyclase, ACG, is post-transcriptionally upregulated in the posterior region of the sorogen. cAMP, produced by ACG, acts on both cARs and PKA to induce the differentiation of prespore cells^2^. A third adenylate cyclase, ACR, acts later on PKA to trigger the maturation of spores and assist in the maturation of stalk cells^3^. ACG acting on PKA has a second role in the mature fruiting body, where it mediates inhibition of spore germination by ambient high osmolyte levels^4^. The cAMP phosphodiesterase RegA also plays a major role in regulating cAMP levels during cell differentiation and spore germination. RegA activity is regulated by signals that are exchanged between the maturing spore and stalk cells. These signals bind to sensor histidine kinases/phosphatases which regulate the phosphorylation state of the intrinsic response regulator of RegA^5^,^6^,^7^.

*D. discoideum* resides in group 4 of the four major groupings of Dictyostelia^8^, which together are members of Amoebozoa, a kingdom that consists mainly of unicellular amoebas that encyst individually. While *D. discoideum* development is strictly multicellular, many dictyostelids in groups 1–3, have retained the ancestral process of encystation, in addition to fruiting body formation, and are thus ideally suited to investigate how cellular communication systems adapted during the transition to multicellularity. One of these species, *Polysphondylium pallidum*, is like *D.discoideum* amenable to both forward and reverse genetic approaches. In addition to the *D. discoideum* genome^9^, the genomes of *P.pallidum*, residing in group 2, *D. fasciculatum*, residing in group 1 and *D. lacteum*, residing in group 3 were recently sequenced by ourselves and colleagues^10^(Schaap, P. and Gloeckner, G. in preparation). Genome sequence of the strictly unicellular amoebozoan *Acanthamoeba castellani* is also available^11^. This information allows us to retrace conservation and change in known developmental signalling genes. We have developed procedures for successive disruption of multiple genes in *Polysphondylium pallidum,* which allows us to assess gene function in both unicellular and multicellular development^12^. In this work we test the hypothesis that multicellular sporulation is evolutionary derived from unicellular encystation by investigating the roles of the catalytic subunit of PKA (PkaC) and the adenylate cyclases ACG and ACR in the uni- and multicellular life cycles of *P. pallidum.*

## Results

### Conservation of PkaC, ACG and ACR in Amoebozoa

**Figure 1:**
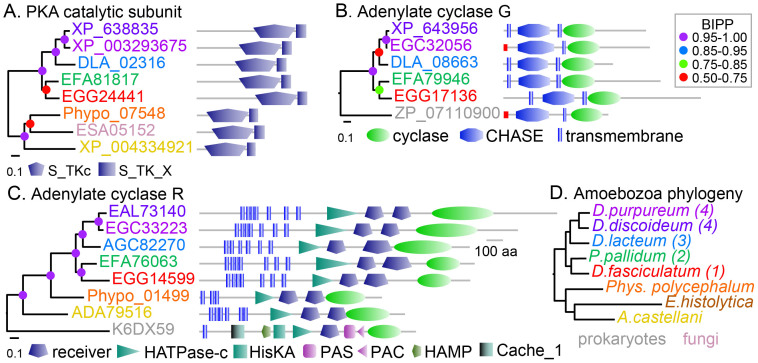
Conservation of PkaC, ACG and ACR in Amoebozoa. (A–C). PkaC, ACG and ACR sequences were retrieved from Dictybase (http://dictybase.org/) or by BLASTp or tBLASTn query of sequenced genomes using *D. discoideum* PkaC*,* ACG and ACR as bait. Sequences were aligned using Clustal Omega^30^ and phylogenetic relationships were determined with MrBayes^31^. The posterior probabilities of tree nodes are indicated by colored dots and the trees are annotated with the functional domain architecture of the proteins, as determined with SMART^32^. Gene identifiers are color-coded to reflect species names as outlined in panel D. (D). Phylogeny of Amoebozoa inferred from 32 aligned protein sequences^33^. Numbers between brackets denote the taxon group in which the Dictyostelid species reside.

Social amoebas can be subdivided into four major groups, which together are members of Amoebozoa, a kingdom of mainly unicellular amoebas^8^. Genomes, representative of the four groups and three genomes of unicellular Amoebozoa have recently become available^9^,^10^,^11^,^13^ (http://sacgb.fli-leibniz.de/; http://www.physarum-blast.ovgu.de/). We investigated the presence of genes encoding ACR, ACG and PkaC, the catalytic subunit of PKA in these genomes. BLAST queries detected homologs of PkaC and ACR in all Amoebozoan genomes, except that of the obligatory parasite *Entamoeba histolytica*, while ACG was only conserved in the Dictyostelid genomes (Figure 1). Because the PkaC sequence is similar to that of many other protein kinases, we also included the closest homologs of PkaC (PkgB and PkgD) in our query. Phylogenetic inference showed that our initally selected PkaC orthologs grouped together with *D. discoideum* PkaC, while hits to the PKA homologs grouped with either PkgB or PkgD (Supplementary Figure 1).

All ACG proteins consist of a CHASE domain flanked by one or two transmembrane domains and a cytosolic adenylate cyclase domain (Figure 1). The ACR homologs also display a similar functional domain architecture as *D. discoideum* ACR with 6–7 transmembrane domains, a HATPase-c domain, two receiver domains and a cyclase catalytic domain. The HisKA autophosphorylation/dimerization domain that is usually located at the N-terminus of the HATPase-c domain is missing in the Amoebozoan ACRs. The closest homologs to ACG or ACR are prokaryote ACs, while a fungal PkaC is closest to the amoebozoan PkaCs.

### Disruption of *P.pallidum* *PkaC* by homologous recombination

**Figure 2:**
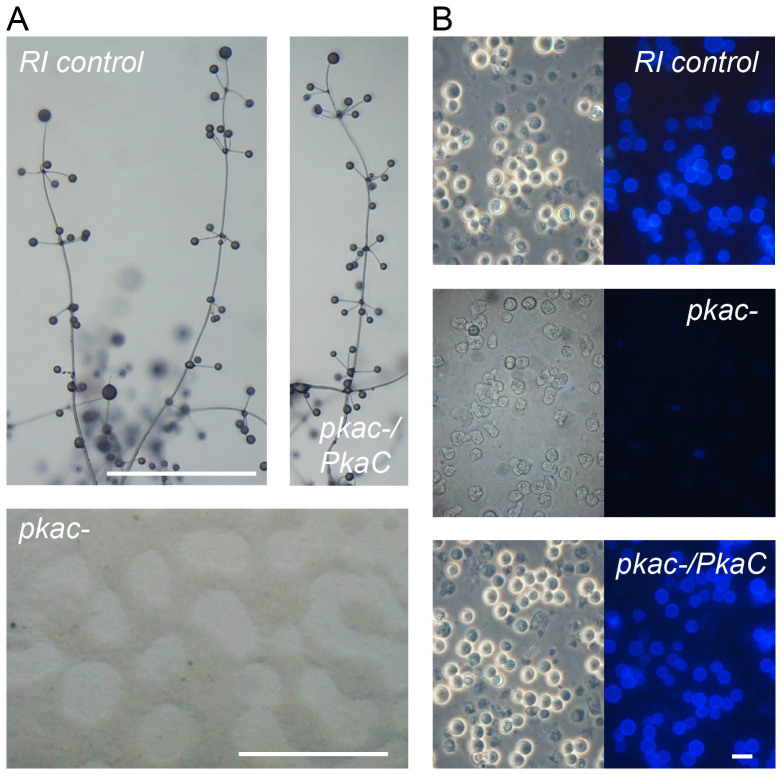
Phenotype of *P.pallidum* *pkac-* and *pkac-/PkaC* mutants. (A). *Fruiting body formation.* Amoebas of the *P. pallidum* random integrant (RI) control, *pkac-* mutant and *pkac-* complemented with *PkaC* and its 5′ intergenic region (see Supplementary figure 2 for generation of mutants), were freed from bacteria, incubated for 48 h on NN agar and photographed. Bar: 1 mM. (B). *Encystation.* RI control, *pkac-* and *pkac-/PkaC* amoebas were incubated in 0.4 M sorbitol for 48 h, stained with 0.001% Calcofluor and photographed under phase contrast and UV illumination (right panels). Bar: 10 μM.

To assess the function of PkaC in uni- and multicellular development, we disrupted *P. pallidum* *PkaC* by replacing a *PkaC* internal segment with a floxed G418 cassette^14^ (Supplementary Figure 2). The *pkac-* amoebas and a control random integrant (RI) proliferated normally, but completely lost the ability to aggregate and form fruiting bodies. After 48 h of starvation, when RI cells had formed fruiting bodies, the lawn of starving *pkac-* cells had somewhat contracted, but aggregates were never formed (Figure 2A). Strikingly, unlike RI controls, the *pkac-* amoebas also could not form cysts when starved at high osmolarity (Figure 2B), a condition that triggers encystation of unicellular amoebas^15^.

After removal of the G418 selection cassette by transformation with cre-recombinase, the *pkac-* cells were transformed with the *PkaC* coding sequence fused to its own promoter. The resulting *pkac-/PkaC* cells fully regained both development into fruiting bodies and encapsulation of single amoebas into cysts (Figs. 2A,B), thus demonstrating that PkaC is essential for both multicellular development and encystation.

### Disruption of *AcgA* and *AcrA* in *P.pallidum*

**Figure 3:**
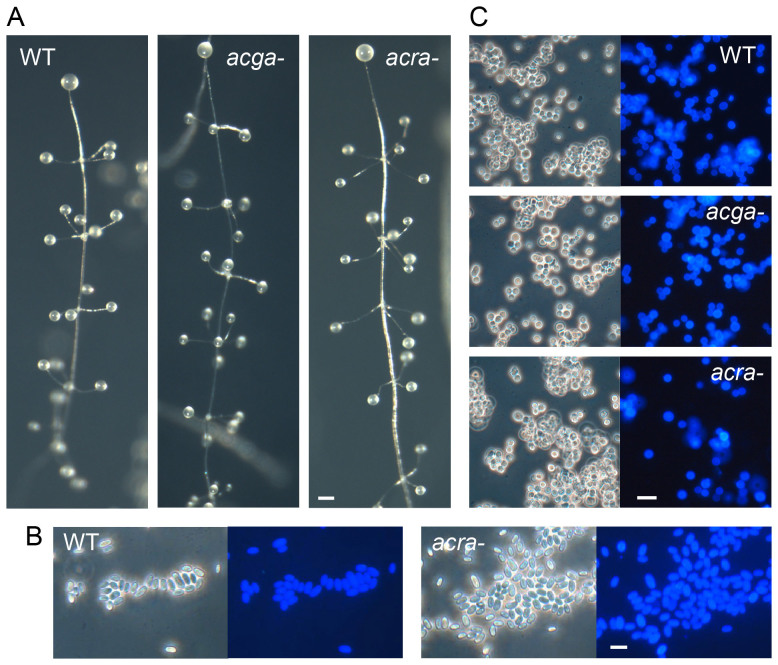
Phenotype of *acga-* and *acra-* mutants. (A). Fruiting body formation. *P. pallidum* wild-type, *acga-* and *acra-* amoebas, freed from bacteria, were incubated on NN agar for 48 h under ambient room lighting and photographed. Bar: 100 μM. (B). Spores. Wild type and *acra-* spores were harvested from fruiting bodies, stained with 0.001% Calcofluor and photographed under phase contrast and UV illumination (right panels). Bar: 10 μM. (C). *Encystation.* Wild-type, *acga-* and *acra-* amoebas, freed from bacteria, were incubated in 0.4 M sorbitol for 48 h, stained with Calcofluor and photographed as above. Bar: 10 μM.

**Figure 4:**
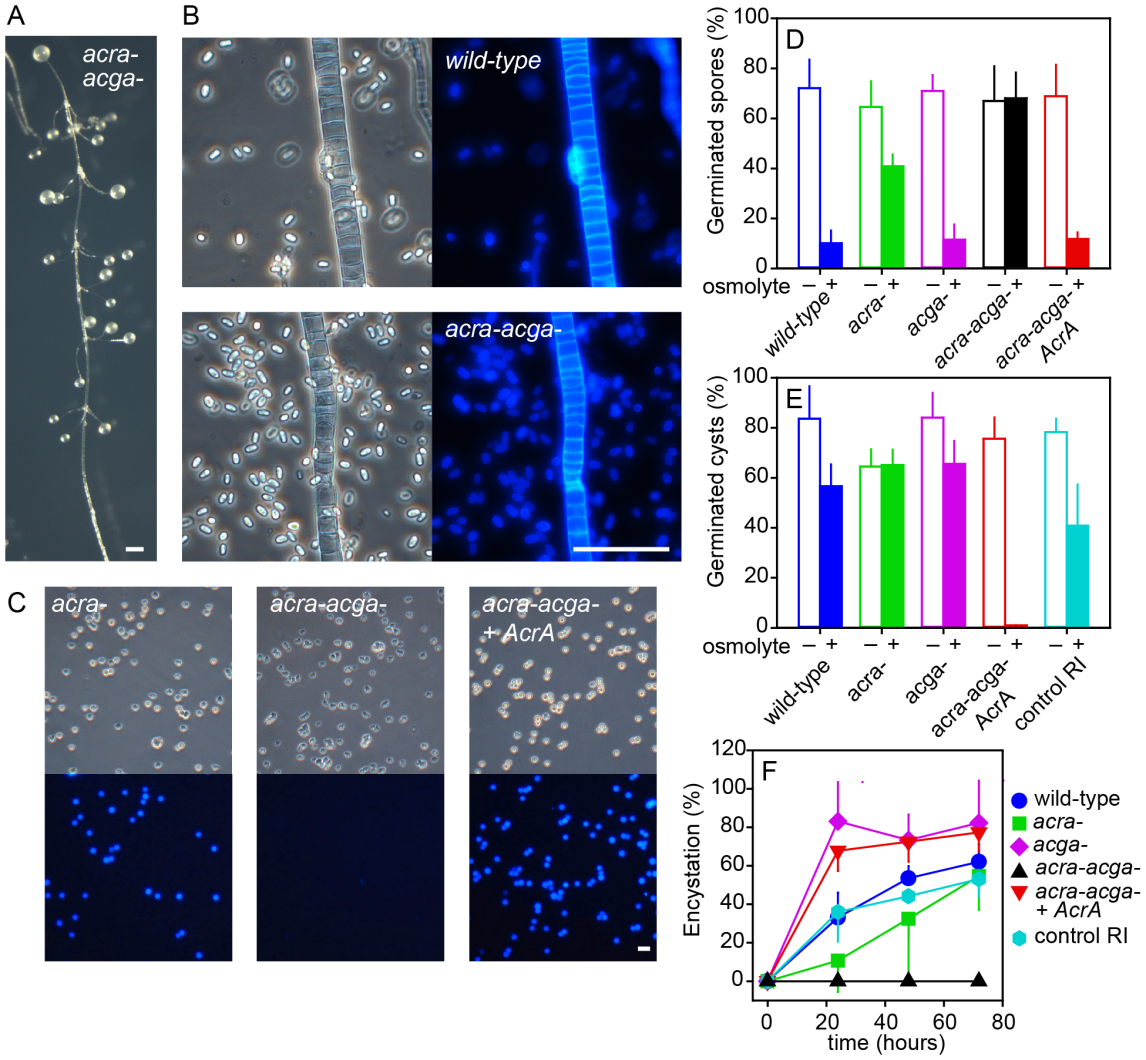
Phenotype of a *P.pallidum acra-/acga-* mutant. (A). *Fruiting body morphology.* The *AcgA* gene was deleted in an *acra-* mutant (see Supplementary figure 3) and the resulting *acra-acga-* cells were developed for 48 h on NN agar. Bar: 100 μm. (B). *Stalk and spores.* Wild-type and *acra-acga-* fruiting bodies were picked up with a needle, deposited in 0.001% Calcofluor on a slide glass and photographed under phase contrast and UV. Bar: Bar 100 μm. (C). *Cysts.* Wild-type, *acra-acga-* and *acra-acga-* cells transformed with *P. pallidum AcrA* and its 5′intergenic region were incubated for 48 h in 0.4 M sorbitol and stained with Calcofluor. Bar: 10 μm. (D). *Spore germination.* Detergent treated spores of wild-type *P. pallidum*, *acra-*, *acga-*, *acra-acga-* and *acra-acga-/AcrA* were plated with *E.coli* on LP agar or LP agar with 0.4 M sorbitol at 200 spores per plate (143 cm^2^). Emerging plaques were counted after 4–6 days at 22°C. Means and SD of 3 experiments with duplicate plates for each strain are presented. (E). *Cyst germination.* Detergent treated cysts of the above cell lines, except *acra-acga-,* which does not form cysts, were plated in the presence and absence of sorbitol as described for spores. In addition to the wild-type, a random integrant of the *AcrA* knock-out construct in wild-type was included as a control. Means and SD of 4 experiments with duplicate plates are presented. (F). *Encystation time course.* Amoebas of the cell lines mentioned above were incubated in KK2 with 0.4 M sorbitol. At the indicated time points, samples were stained with Calcofluor and the numbers of fluorescent cysts and unstained amoebas were counted. Means and SD of 4 experiments, performed in duplicate.

ACG has an overlapping role with ACR in prespore differentiation in *D. discoideum* and mediates inhibition of spore germination by high ambient osmolarity in the spore head^2^,^4^,^16^. To investigate whether ACG has essential roles in *P. pallidum* multicellular development and encystation, we disrupted the ACG gene *AcgA* in *P. pallidum* (Supplementary figure 3). The *acga-* mutant formed normal aggregates and fruiting bodies (Figure 3A). Surprisingly, the fruiting bodies contained viable detergent resistant spores, that showed normal inhibition of spore germination by high osmolarity (Figure 4D). The *acga-* mutant also showed normal encystation (Figure 3C).

ACR is essential for spore maturation in *D. discoideum*^3^ and to assess its role in *P. pallidum*, we next ablated the *P. pallidum AcrA* gene (Supplementary Figure 4). Surprisingly, unlike *D. discoideum* *acra-*, the *P. pallidum* *acra-* mutant displayed normal spore formation in fruiting bodes (Figure 3A,B). The *acra-* mutants also encysted when submerged at high osmolarity, but encystation was less efficient than in wild-type *P. pallidum* (Figs. 3B, 4F). To assess whether ACG and ACR are functionally redundant, we created a double *acra-acga-* mutant. *P. pallidum acra-* cells were transformed with cre-recombinase to excise the neomycin selection cassette and G418 sensitive clones were selected, which were then transformed with the *AcgA* knock-out construct. Strikingly, the *acra-acga-* mutant was also not defective in fruiting body morphology or sporulation (Fig. 4A,B). However, the ability to encyst was completely lost (Fig. 4C). To validate that this was due to loss of cAMP synthesis, the neomycin cassette was excised from the *acra-acga-* mutant once more, and cells were transformed with a plasmid that contained a *P. pallidum* genomic fragment, encompassing the full length *AcrA* coding and 5′intergenic region. The resulting *acra-acga-/AcrA* mutant regained the ability to encyst (Fig. 4C), confirming that lack of encystation was due to the combined loss of *AcgA* and *AcrA*.

### Roles for ACG and ACR in regulation of encystation and spore germination by osmolytes

When comparing *P. pallidum* to *D. discoideum*, a striking difference is the absence of an obvious role for ACR or ACG in *P. pallidum* spore differentiation. It is also remarkable that ACR can complement ACG in osmolyte-induced encystation, a role more suited for ACG with its intramolecular osmosensor^17^. In *D. discoideum*, high osmolarity prevents spores from germinating prematurely, while still in the sorus. We first investigated whether ACG or ACR still regulated spore germination in *P. pallidum*. Mature spores from wild-type and mutant fruiting bodies were incubated with detergent to lyse unencapsulated cells, and plated clonally on *E.coli* lawns in the presence and absence of 0.4 M of the osmolyte sorbitol. In the absence of osmolyte, wild-type, *acra-, acga-, acra-acga-* and *acra-acga-/AcrA* spores germinated with equal efficiency (Figure 4D). In the presence of osmolyte, germination of wild-type, *acga-* and *acra-acga-/AcrA* spores was about 85% reduced and of *acra-* spores about 30%. Only *acra-acga-* spores still germinated at full efficiency. These data show that ACG and ACR both mediate inhibition of spore germination by high osmolarity, with ACR playing the more dominant role.

Cyst germination on *E.coli* lawns is only 30% reduced by 0.4 M sorbitol in wild-type *P. pallidum* and by about 50% in a random integrant of the *AcrA* KO construct (Fig. 4E). Inhibition of cyst germination by osmolyte was completely lost in the *acra-* mutant, but not in the *acga-* mutant. Remarkably, cyst germination was completely inhibited when *acra-* cells were complemented with *AcrA* expressed from its own promoter. This could be a consequence of *AcrA* overexpression, caused by integration of multiple copies of the expression vector in the *P. pallidum* genome.

A time course of the progression of encystation in wild type *P. pallidum* and all adenylate cyclase mutants showed that 60% of wild-type and RI control amoebas have encysted after 72 h of incubation with osmolyte (Fig. 4F). The *acra-acga-* cells have completely lost encystation, while *acra-* cells encyst more slowly. Unexpectedly, both the *acga-* cells and the *acra-acga-/AcrA* cells reached 70–80% encystation within 24 hours. Because *acra-acga-/AcrA* also showed more efficient inhibition of cyst germination by high osmolarity, this may simply imply that the increased ACR levels facilitated encystation. Why ACG should be required for encystation in an *acra-* background, but simultaneously reduce encystation in a wild-type background is less clear. It suggests a scenario in which ACR is the main player, with its expression down-regulated by ACG. Negative regulation of *AcrA* expression by ACG was previously observed in *D.discoideum* slugs^2^.

## Discussion

ACR and ACG have overlapping roles in inducing prespore differentiation and spore maturation in *D.discoideum*, but appeared to be dispensible for the same processes in *P.pallidum* spore differentiation. However, the *P. pallidum* genome contains three copies of the gene encoding the adenylate cyclase ACA^10^, which is mainly involved in chemotactic signalling in *D.discoideum*^7^. One of the *Ppal* copies, ACA2, is expressed in prespore cells (Kawabe, Y., in preparation) and likely provides cAMP for spore differentiation.

The most striking outcome of this work is that deletion of *PkaC* and combined deletion of *AcrA* and *AcgA* completely blocks the process of encystation in *P. pallidum*. PkaC and ACR, but not ACG, are conserved in other Amoebozoa, such as *Acanthamoeba castellani* and *Physarum polycephalum* (Figure 1). Other components of the cAMP signalling pathway such as PkaR and the cAMP phosphodiesterase RegA are also conserved in these amoebozoan genomes and, remarkably, also in the amoeboflagellate *Naegleria gruberi*, which resides in the kingdom Excavata^11^,^18^,^19^.

RegA plays a central role in regulating cAMP levels during *D. discoideum* multicellular development, where its activity is controlled by intercellular signalling. The signals bind to sensor histidine kinases/phosphatases (SHKPs), which regulate the phosphorylation state of the intrinsic response regulator of RegA and thereby the attached cAMP phosphodiesterase activity^7^. The genomes of *Naegleria* and all sequenced Amoebozoa contain a large number of SHKPs. In non-dictyostelid amoebas, the roles of SHKPs have not yet been explored, but in *D. discoideum* their main target is RegA. About 7 out of the 15 *Dictyostelium* SHKPs mediate effects of secreted factors that control the developmental programme. However the stimuli detected by the other 8 SHKPs and those of the unicellular amoebas are likely to be of environmental origin.

**Figure 5:**
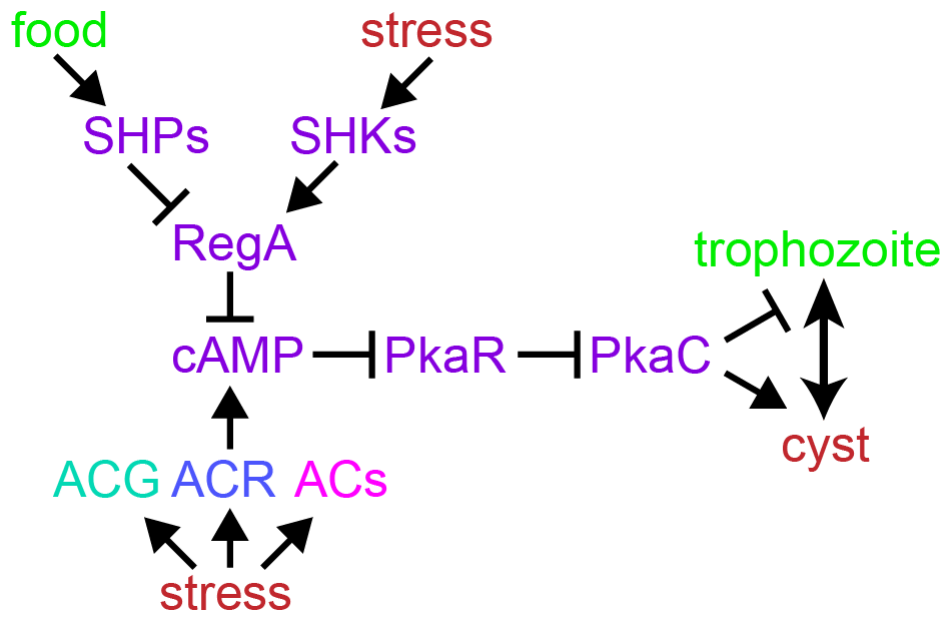
Pathway for encystation in Amoebozoa and Excavata. The choice between feeding trophozoite and dormant cyst stage is controlled by cAMP binding to PkaR causing dissociation from and activation of PkaC. cAMP is synthesized by adenylate cyclases, which may be activated directly by stress. cAMP is hydrolyzed by the cAMP phosphodiesterase RegA, which is respectively activated by sensor histidine kinases that sense food, or inhibited by sensor histidine phosphatases that sense stress. Violet: components that are conserved in Amoebozoa and Excavata, blue: conserved in Amoebozoa, Teal or pink, conserved in only dictyostelia or Excavata, respectively. Arrow: stimulates; crossbar: inhibits.

Loss of RegA causes precocious encystation in *P. pallidum* and *A.castellani*^19^, as expected for a protein that inhibits PKA function*.* Combined with results obtained in this work, this allows us to propose a universal mechanism for control of amoebozoan and excavate encystation (Figure 5). In this scheme, cAMP activation of PKA induces encystation and prevents excystation. cAMP is synthesized by ACR in Amoebozoa, while in both Amoebozoa and Excavata, cAMP is hydrolysed by RegA. RegA activity is under both positive and negative regulation from external stimuli that activate a sensor histidine kinase or a sensor histidine phosphatase, respectively. For the former, such stimuli could be stress factors that cause encystation and for the latter, the stimuli could signal the presence of food to cause cyst germination.

Researchers have been intrigued for many years by the fact that PkaC regulates so many aspects of *D. discoideum* development, starting with the transition from growth to development^20^, the regulation of chemotactic signalling^21^, the differentiation of prespore cells^22^, the maturation of spores and stalk cells^23^,^24^,^25^ and the germination of spores^4^. We show here that these roles are likely to have emerged from an original role of PKA in mediating stress-induced encystation and stress-inhibited excystation. The trigger for multicellular development is also nutrient stress and its end-point is the formation of two encapsulated cell types, the stalk cells and spores. It therefore appears that PKA retained its ancestral role in encapsulation, when Dictyostelia started to adopt a multicellular survival strategy, but additionally acquired novel roles to assist the organism to proceed through its multicellular differentiation programme in a well-regulated manner. These novel roles required novel mechanisms to regulate cellular cAMP levels. The differences in ACG, ACR and ACA function that we noted in this work between the group 2 species *P.pallidum* and the group 4 species *D.discoideum* may reflect different evolutionary trajectories towards effective cell signalling between these groups.

In conclusion, we propose that the range and complexity of the cAMP-mediated signalling cascades that control almost all stages of multicellular development in modern Dictyostelia have been co-opted from an ancestrol role for cAMP in stress-induced dormancy. The emergence of complex cell-cell communication from basic environmental sensing in Dictyostelia may prove to be a paradigm for the evolution of multicellularity in other eukaryote lineages.

## Methods

### Cell growth, development, encystation and sporulation

#### Growth and development

*Polysphondylium pallidum* strain PN500, was routinely grown in association with *Escherichia coli* on lactose peptone (LP) agar. For multicellular development, *P. pallidum* cells were harvested in 20 mM KH_2_PO_4_/K_2_HPO_4_, pH 6.5 (KK2), washed free from bacteria and incubated on non-nutrient (NN) agar at 10^6^ cells/cm^2^ and 22°C.

#### Encystation

For quantification of encystation, *P. pallidum* cells were grown in a suspension of autoclaved *Klebsiella* *aerogenes* in KK2_,_ until cell density reached 5 × 10^6^–10^7^ cells. Cells were washed, resuspend in KK2 with 0.4 M sorbitol at 10^7^ cells/ml and shaken at 180 rpm and 21°C. Aliquots of 0.1 ml were sampled at regular intervals and supplemented with 1 μl 0.1% Calcofluor (which reacts to cellulose in the cyst wall). Total amoeba and cyst numbers were determined by counting cells in a haemocytometer under phase contrast and UV illumination, respectively. 100–500 cells were counted for each time point.

#### Cyst and spore germination

*P. pallidum* spores were harvested from 5-day old fruiting bodies. For cysts, *P. pallidum* cells were grown in *K.aerogenes* suspension as described above, washed and incubated with 0.4 M sorbitol for 3 days to allow mature cysts to form. Cysts or spores were treated for 10 min with 0.1% Triton-X100 to lyse amoeboid cells, washed with KK2, counted and clonally plated on LP with *E. coli.* Emerging *P. pallidum* plaques were counted after 4–6 days.

### Gene disruption and complementation

#### Disruption of P. pallidum PkaC

For disruption of *PkaC*, two fragments of *P. pallidum* gDNA were amplified using primer pairs PKACI5′/PKACI3′ and PKACII5′/PKACII3′ (Supplementary table 1). The fragments were digested with XbaI/BamHI or XhoI/KpnI, using restriction sites incorporated in primer design, and sequentially inserted into inserted into XbaI/BamHI and XhoI/KpnI digested plasmid pLox-NeoIII^14^ to flank the LoxP-neo selection cassette (Supplementary figure 2A). *P. pallidum* cells were transformed by electroporation with the XbaI/KpnI vector insert, followed by selection of transformants on autoclaved *K.aerogenes* at 300 μg/ml G418^26^. Genomic DNA was isolated from G418 resistant clones and screened by 2 PCR reactions (Supplementary figure 2B) and Southern blot (Supplementary figure 2C) to diagnose *PkaC* disruption by homologous recombination. Two knock-out (KO) and two random integrant (RI) clones were identified.

#### Complementation of pkac- with PkaC

To remove the A6neo cassette, KO (*pkac-*) cells were transformed with pA15NLS.Cre^27^ for transient expression of Cre recombinase. Transformed clones were replica-plated onto autoclaved *K.aerogenes* on LP agar with and without 200 μg/ml G418 for negative selection. To generate a *PkaC* expression construct, the 0.8 kb *PkaC* 5′intergenic region was amplified from gDNA using primers P-PKAC p3 and P-PKAC P4r (Supplementary table 1), which contain NheI and BglII restriction sites, respectively, and ligated into NheI/BglII digested vector pDdCGFP^28^. Next, the *PkaC* coding sequence was amplified using primers palPKAC p9 and palPKAC p11r (Supplementary table 1), which harbour BglII and XbaI sites, respectively, and ligated into the BamHI and XbaI sites of the newly created vector. This fuses *PkaC* N-terminally to its own 5′intergenic region and C-terminally to green fluorescent protein (GFP)(see Supplementary figure 2A). The construct was transformed into *pkac-* cells and G418 resistant cells were selected as described above.

#### Disruption of P. pallidum AcgA

To disrupt *P. pallidum AcgA*, two *AcgA* fragments of 2.8 and 1.1 kb, respectively, were amplified from *P. pallidum* gDNA using primer pairs Pp-ACG-P5/Pp-ACG-P3 and Pp-ACG-53H/Pp-ACG-53K (Supplementary table 1). The first fragment was reduced to 2.4 kb by XbaI/BglII digest and inserted into XbaI/BamHII digested vector pLox-NeoIII^14^. The second fragment was digested with HindIII/KpnI, using restriction sites introduced in the primers, and inserted into the HindIII/KpnI sites of the newly generated vector pAcgA-KO (Supplementary figure 3). *P. pallidum* cells were transformed by electroporation with the linearized vector pAcgA-KO, followed by selection of transformants and diagnosis of gene knock-out as outlined in Supplementary figure 3.

#### Disruption of P. pallidum AcrA

For *P. pallidum* *AcrA* disruption, two DNA fragments of ~2.0 kb were amplified by PCR from *P. pallidum* gDNA, using primer pairs AcrA5′-fw/AcrA5′-rev and AcrA3′-fw/AcrA3′-rev (Supplementary table 1), which harbour KpnI/BamHI and HindIII restriction sites, respectively. The two fragments were sequentially cloned into the KpnI/BamHI and HindIII restriction sites of plasmid pLoxP-NeoI^29^, yielding vector pAcrA-KO. Correct orientation of the HindIII fragment was verified by digest with SalI and XbaI and DNA sequencing. The insert containing the two *AcrA* KO fragments flanking the floxed neomycin resistance cassette (Supplementary figure 4A) was introduced into *P. pallidum* cells, and gene knock-outs were identified by PCR and Southern blot (Supplementary figure 4B,C).

#### Creation of a double acra-acga- cell line

The A6neo cassette was removed from *AcrA* KO cells (*acra-*) as described above. A G418 sensitive *acra-* clone was transformed with linearized pAcgA-KO to delete *AcgA*. The *AcgA* gene disruption was confirmed by PCR and Southern blot (Supplementary figure 3C).

#### Complementation of acra-acga- with AcrA

To express *AcrA* from its own promoter, a fosmid, used for *P. pallidum* genome mapping^10^ and containing *AcrA*, was digested whith SpeI. This released an 8.65 kb fragment, which contained the entire *AcrA* 5′intergenic region and coding sequence. This fragment was cloned into vector pLox-NeoIII and introduced into *acra-acga-* cells.

## Author Contributions

Y.K. prepared the *acga-*, *acra-acga* and *acra-acga-/AcrA* mutants, C.S. the *acra-* mutant and Q.D. the *pkac-* and *pkac-/PkaC* mutants. All three examined the phenotypes of their mutants and wrote sections of the manuscript. P.S. designed the study and finalized the manuscript.

## Supplementary Material

Supplementary Information Supplementary Information
