# Supplementary material for: A Conserved Signalling Pathway for Amoebozoan Encystation that was Co-Opted for Multicellular Development
Source: Sci Rep. 2015 Apr 16;5:9644. doi: 10.1038/srep09644 (PMC4399386; doi:10.1038/srep09644)
Supplement: Supplementary Information [file srep09644-s1.doc]

Supplementary Information

A conserved signalling pathway for Amoebozoan encystation that was co-opted for multicellular development

Yoshinori Kawabe, Christina Schilde, Qingyou Du and Pauline Schaap

Supplementary figure 1. Phylogeny of Dictyostelid PkaC orthologs and paralogs

**
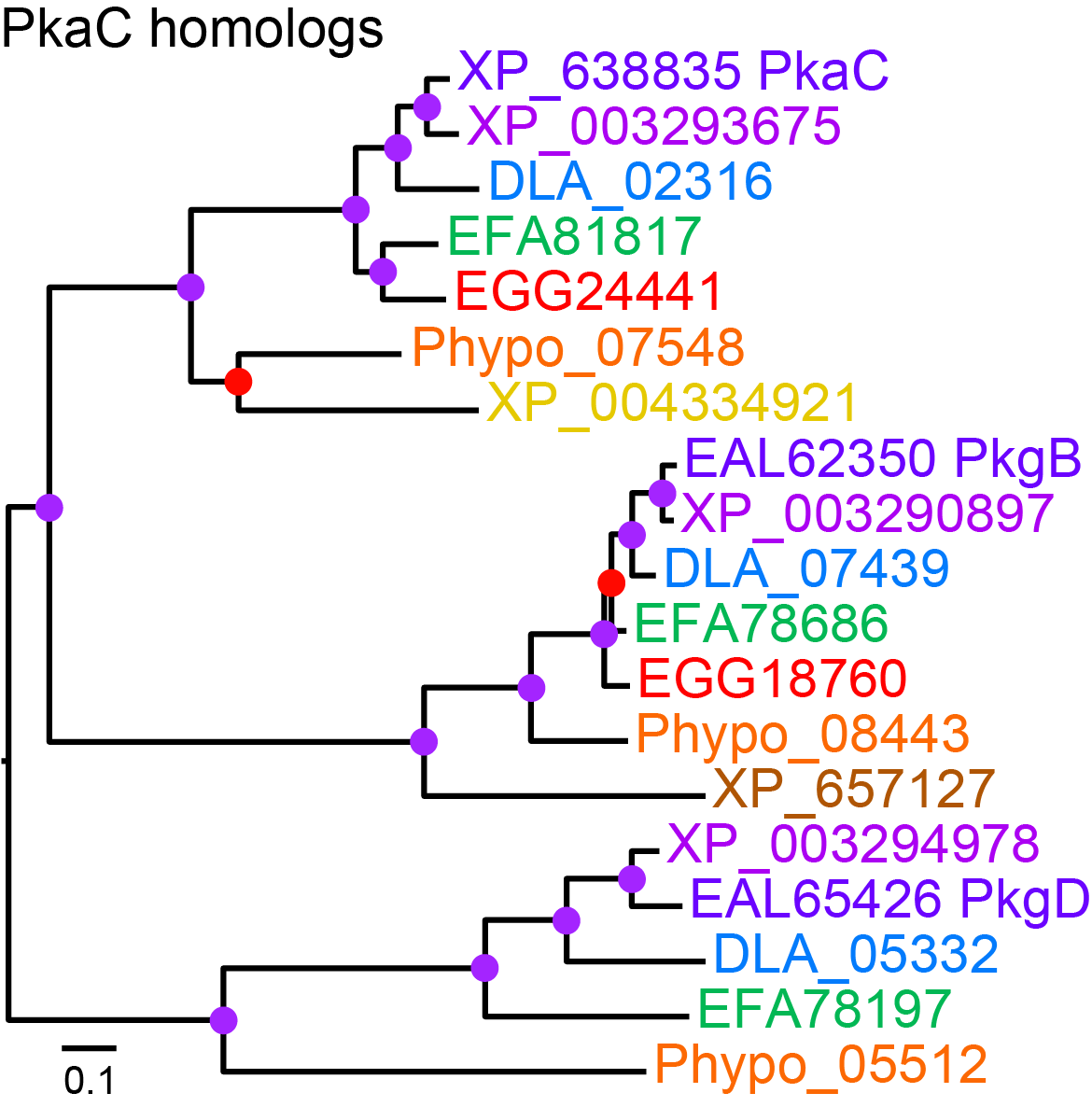
**

PkaC and its first BlastP hits in *D.discoideum*, PkgB and pkgD, were used as bait to query the sequenced genomes of other Amoebozoa. The best bidirectional hits for each query were aligned using ClustalOmega with five iterations. Regions of sequence that were not ambiguously aligned were deleted. The edited alignment was subjected to Bayesian phylogenetic inference using a mixed amino acid model with rate variation between sites estimated by a gamma distribution with a proportion of invariable sites. The analysis was run for 10^5^ generations at which point the SD of split frequencies was 0.009. The tree is rooted at midpoint. Genbank accession numbers are color-coded to reflect species as in figure 1 main text. The color coding of node probabilities is also the same as in figure 1.

Supplementary Figure 2. *P.pallidum PkaC* gene model, expression construct, knock-out strategy and diagnosis


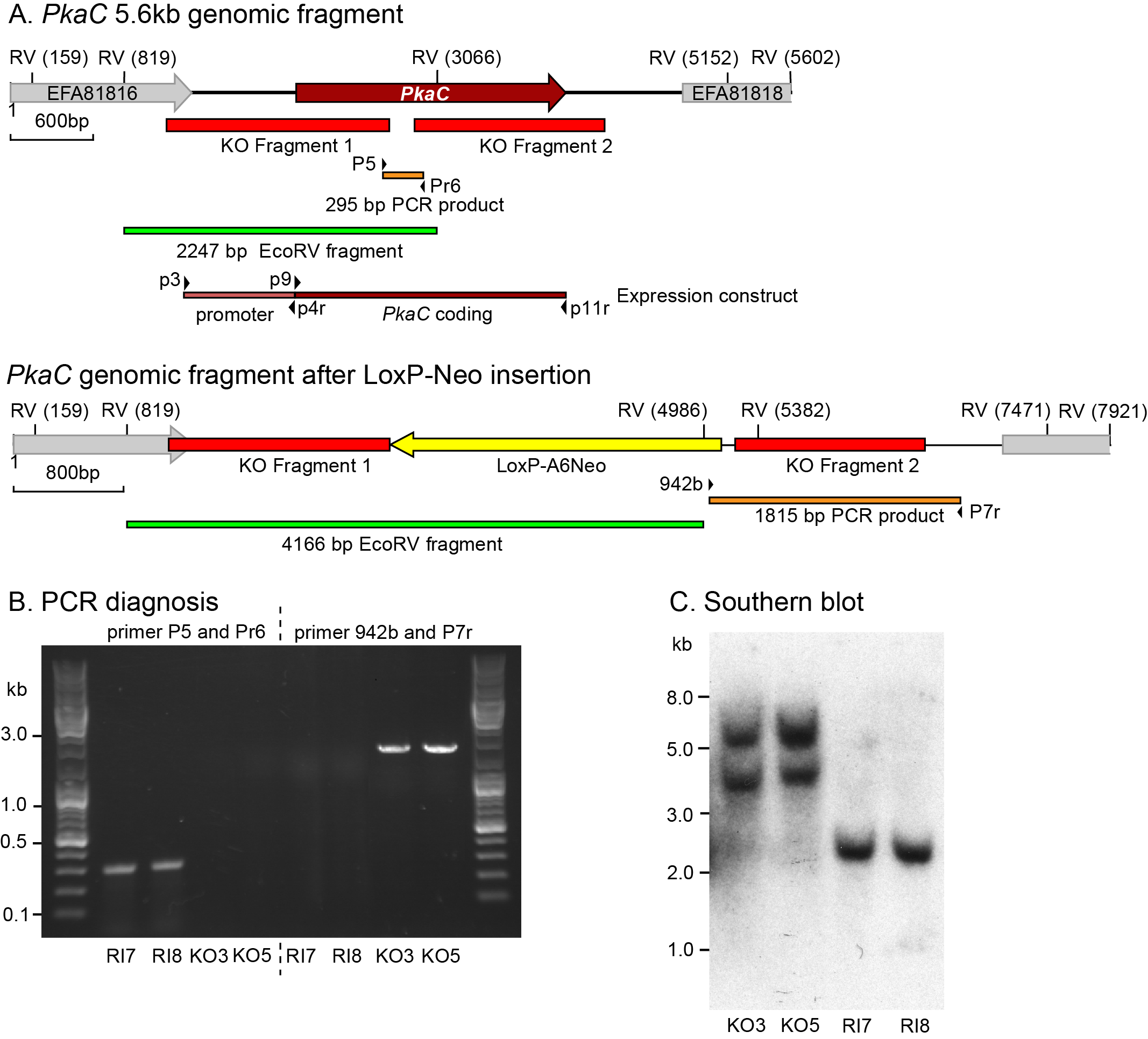


A. *Gene model.* Model of a 5.6 kb genomic fragment that harbours the *PkaC* gene and two flanking genes before and after insertion of the loxP-A6Neo cassette by homologous recombination. For gene disruption, KO fragments 1 and 2 were amplified from *P.pallidum* gDNA and inserted into XbaI/BamHI and XhoI/KpnI sites of pLoxNeoIII to flank the LoxP-A6Neo cassette. The linearized construct was introduced into *P. pallidum* cells and G418 resistant clones were identified (See Methods for details). The positions of primers used to diagnose gene disruption or to generate a *PkaC* expression construct are indicated, together with their amplified products.

B. *PCR knock-out diagnosis.* Genomic DNAs were isolated from G418 resistant clones and subjected to PCR, using primer pair PkaC P5/Pr6, which should yield a 295 bp product with intact *PkaC,* and primer pair 942b/P7r (Table S1), which yields a 1815 bp product, when the Loxp-Neo cassette is inserted in the *PkaC* locus. The analysis shows that clones 7 and 8 harbour random vector integrations (RI), while clones 3 and 5 are knock-outs (KOs).

C. *Southern blot diagnosis.* Genomic DNAs from *c*lones 3,5,7 and 8 were digested with EcoRV, size-fractionated on agarose and transferred to nylon membranes. The Southern transfer was probed with ^32^P-labeled KO fragment 1 obtained by XbaI/BamHI digestion. In wild-type and random integrants the probe should hybridize to a 2.25 kb and after loxP-neo insertion to a 4.17 kb fragment (panel A). The larger bands in the KO clones probably result from incomplete gDNA digestion.

**Supplementary Figure 3. *P.pallidum AcgA* gene model, knock-out strategy and diagnosis**


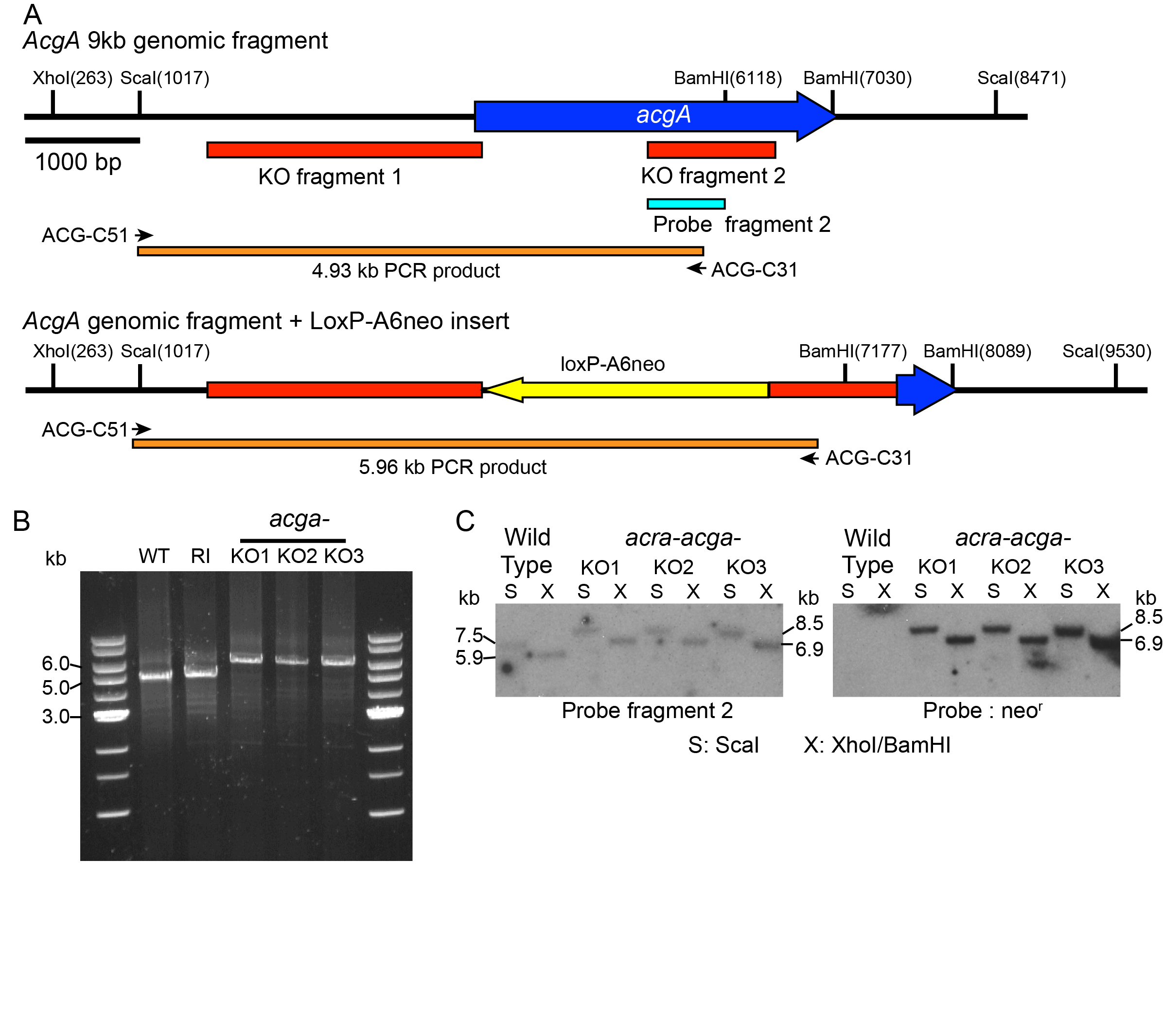


A. *Gene model.* Model of a 9 kb genomic fragment that harbours the *AcgA* gene before and after insertion of the loxP_A6Neo cassette by homologous recombination. A gene disruption construct was generated by inserting KO fragments 1 and 2, amplified from *Ppal* genomic DNA into vector pLoxNeo-III to flank the LoxP-A6Neo cassette. The linearized construct was introduced into *P. pallidum* cells and G418 resistant clones were identified (See Methods for details).

B. *Diagnosis of AcgA knock-out.* Genomic DNAs were isolated from wild-type and G418 resistant cells and subjected to PCR using primers ACG-C51 and ACG-C31 (Table S1). Positions of primers are shown in Figure A (Arrow heads). 4.93 kb fragments were amplified in wild type (WT) and a transformant with an off-target integration (RI), while 5.96 kb fragments were amplified in three *acga* knock-out clones.

C. *Southern blot of acra-/acga-.* An *acra-* mutant (see figure S5) was transformed with the *AcgA* knock-out construct and after an inital PCR diagnosis, genomic DNAs from wild type and three putative *acra-acga-* KO clones were digested with BamHI/XhoI or ScaI, size-fractionated on agarose and transferred to nylon membranes. The Southern transfer was probed with the ^32^P-labeled neo^r^ gene and (after stripping) with half of KO fragment 2 (see panel A). The insertion of the LoxP_A6Neo cassette in the *AcgA* gene results in a size increase of the BamHI/XhoI fragment from 5.9 to 6.9 kb and of the ScaI fragment size from 7.5 to 8.5 kb with the KO fragment 2 probe. The neo^r^ probe hybridizes to the same bands as KO fragment 2 in the KO clones, but does not hybridize to WT DNA.

**Supplementary Figure 4. *P.pallidum AcrA* gene model, knock-out strategy and diagnosis**


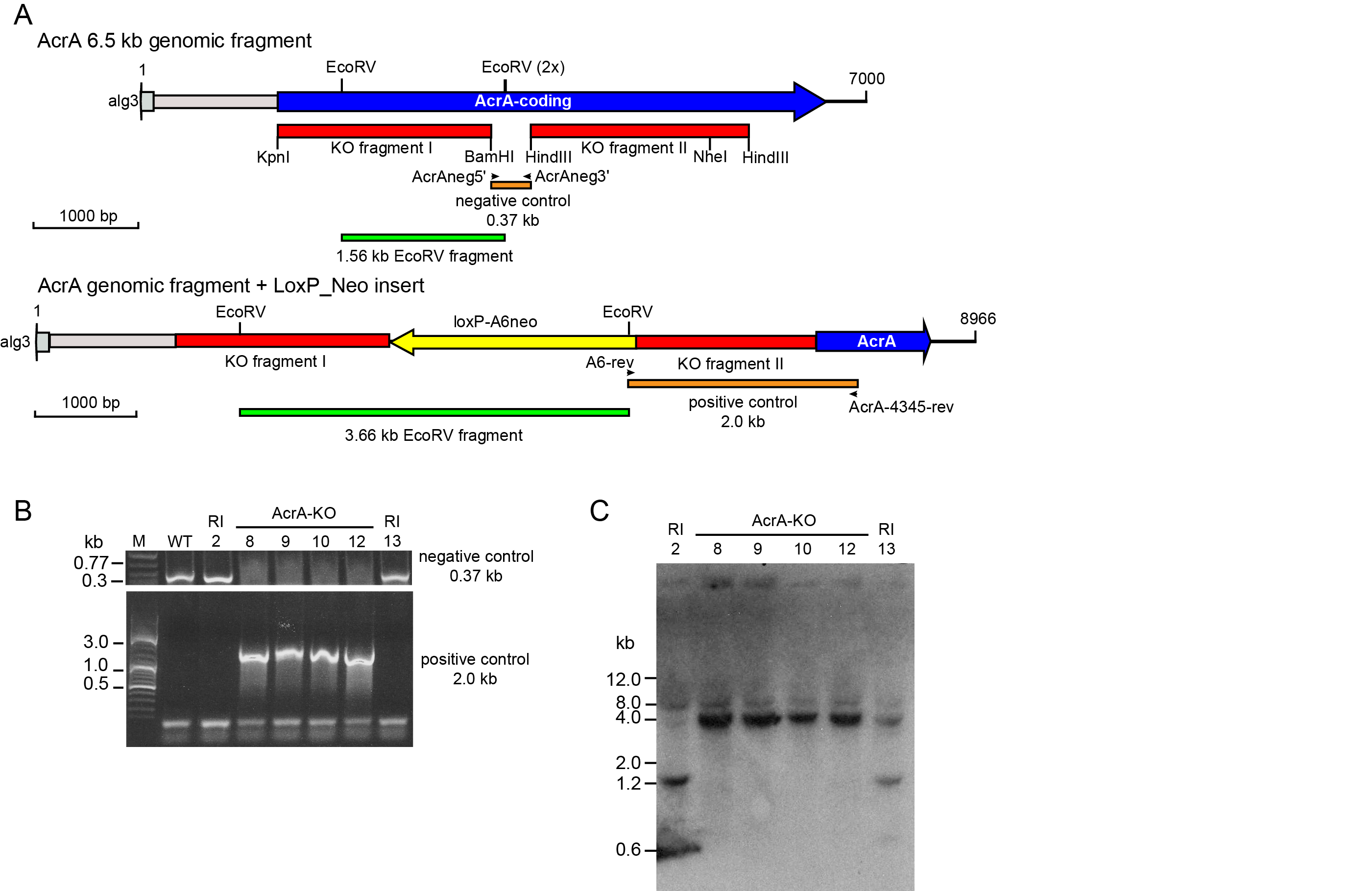


A. *Gene model.* Model of the 7 kb genomic fragment containing the *AcrA* gene before and after insertion of the LoxP_A6Neo cassette. A gene disruption construct was generated by inserting KO fragments 1 and 2, amplified from *Ppal* genomic DNA into vector pLoxNeoI. The segment containing *AcrA* KO fragments flanking the LoxP-A6Neo cassette was introduced into *P. pallidum* cells and G418 resistant clones were identified (See Methods for details).

B. *PCR diagnosis of AcrA knock-out*. After selection for growth at 300 µg/ml G418, transformed clones were screened for homologous recombination by two PCR reactions using primers AcrAneg5’/AcrAneg3’ (negative control) and primers A6-rev/AcrA-4345-rev (positive control) (Table S1). The negative control should yield a 0.37 kb product in wild type and random integrant (RI) cells, but not in knock-out (KO) clones, while the positive control should yield a 2.0 kb product in KO clones.

C. *Southern blot*. Genomic DNAs from several *AcrA* KO and RI clones were digested with EcoRV, size fractionated by electrophoresis, transferred to nylon membrane and probed with ^32^P-labeled fragment 1 of the pAcrA-KO vector. Wild-type cells and random integrants should yield a band at 1.56 kb, which is lost in KO cells. The weak larger hybridisation at 8 kb, which is present in both RI and KO clones, is probably due to an EcoRV site further upstream of KO fragment 1 and weak hybridisation with the 5’-end of KO fragment 1.

**Supplementary Table 1. Oligonucleotide primers used in this work**

| Name | Restriction site | Sequence |
| --- | --- | --- |
| PKACI5’ | XbaI | 5’-GATTCTAGAGTGTGAATGAGAATGGA-3’ |
| PKACI3’ | BamHI | 5’-TGAGGAGCGGATCCGGCAAGA-3’ |
| PKACII5’ | XhoI | 5’-GATCTCGAGTCAAACTCAAACAAGTCG-3’ |
| PKACII3’ | KpnI | 5’-GATGGTACCTATTCATACAAACTTAGG-3’ |
| PkaC P5 |  | 5’-CTACTCTACCATCGATGTCGT-3’ |
| PkaC P6r |  | 5’-AAAAGGATGATGAATTGATGCC-3’ |
| 947b |  | 5’-GGGCAAATCTGTAATTTTCAG-3’ |
| PkaC P7r |  | 5’-CATTGGGTATACACAAATTTAT-3’ |
| P-PKAC p3 | NheI | 5’-GATGCTAGCGCCATAAAGTTAAATAAAA-3’ |
| P-PKAC P4r | BglII | 5’-GATAGATCTTTAATTTATATTTATTTATATTTC-3’ |
| palPKAC p9 | BglII | 5’-GATAGATCTATGAATTATGATTATAATAATAATG-3’ |
| palPKAC p11r | XbaI | 5’-GATTCTAGAAAAATCTCTAAATAGATGAGCATA-3’ |
| Pp-ACG-P5 |  | 5’-GGACTAGTACCTCTACATATGAGTGCATC-3’ |
| Pp-ACG-P3 |  | 5’-GTCAGAGCTGACCTTCTC-3’ |
| Pp-ACG-53H | HindIII | 5’-CCCAAGCTTCTAGAGTCTGGCGAGGATGTCGT-3’ |
| Pp-ACG-53K | KpnI | 5’-GGGGTACCGGTTGTGGATCGGCACTACTAGG-3’ |
| ACG-C51 |  | 5’-CTACTGCAAACAAATGACC-3’ |
| ACG-C31 |  | 5’-TAGTCTCCAGAATGTCCA-3’ |
| AcrA5’-fw | KpnI | 5’-GGGGTACCGGGAAAATCAATTTAATAAGAGGA-3’ |
| AcrA5’-rev | BamHI | 5’-CGGGATCCGCTAATATCAATTTTATATCATCAA-3’ |
| AcrA3’-fw | HindIII | 5’-CCCAAGCTTGGATGACCAACTCAAGATTATCAA-3’ |
| AcrA3’-rev | HindIII | 5’-CCCAAGCTTCGCTGGAATAGCAAATCCCAA-3’ |
| AcrAneg3' |  | 5’-ACTCCAATATCTCCTTTCTCTG-3’ |
| AcrAneg5' |  | 5’-CACTTTAACCAACCAACCAAC-3’ |
| AcrA-4345-rev |  | 5’-CGCCAATGAATTTGTCGATGAA-3’ |
| A6-rev | ApaI | 5’-GGGCCCCACCGTGGTTAATTAATTAACCCGGGAA-3' |
